# Ace in the hole: playing cards show the role of order and magnitude in the SNARC effect

**DOI:** 10.1007/s00426-026-02288-4

**Published:** 2026-04-07

**Authors:** Serena Mingolo, Valter Prpic, Krzysztof Cipora, Eleonora Bilotta, Alberto Mariconda, Tiziano Agostini, Mauro Murgia

**Affiliations:** 1 Department of Physics, University of Calabria, Rende, Cosenza Italy; 2 Department of Life Sciences, University of Trieste, Trieste, Italy; 3 Department of Theoretical and Applied Sciences, eCampus University, Novedrate, Italy; 4 Copernicus Center for Interdisciplinary Studies, Jagiellonian University in Krakow, Krakow, Poland; 5 Centre for Mathematical Cognition, Loughborough University, Loughborough, UK

## Abstract

The SNARC effect indicates that numbers are mapped from left to right as in a mental number line. Accumulating evidence suggests that it could be attributed both to the magnitude or to the order of numbers, but the role of these two aspects has not yet been disambiguated since the two are tightly correlated. This study investigated the influence of order and magnitude in the SNARC effect using playing cards as stimuli. While most people organize cards in ascending order (AO), according to the Western reading-writing direction, a minority of people arrange them in descending order (DO). In this regard DO people should spontaneously associate low magnitude cards (e.g., 2) to the right, and high magnitude cards (e.g., 6) to the left. Therefore, in DO individuals, cards’ order would elicit a spatial mapping opposite to the canonical mapping of quantities (i.e., mental number line). In Experiment 1, DO participants performed magnitude classification on simple numerals and on playing cards, showing a regular SNARC effect when classifying numbers and no significant effect when classifying cards. Conversely, in Experiment 2, AO participants showed a regular SNARC effect when classifying both numbers and cards. In Experiment 3, a separate group of DO participants was recruited and tested online to replicate Experiment 1 and clarify the occurrence of spatial associations in card classification. Results indicated that DO participants showed regular SNARC effects both in number and card classification, suggesting that magnitude played a key role overruling the order of cards. This is apparently in contradiction with the view that specific experiences with ordered stimuli should determine the direction of an association.

## Introduction

Cognitive psychology has widely investigated how people represent abstract concepts in their minds. An abstract activity such as number processing is tightly correlated with spatial representation. Such spatial coding of numbers is demonstrated by a well-known phenomenon named Spatial-Numerical Association of Response Codes (SNARC) effect (Dehaene et al., 1993). Due to this effect, participants who respond to centrally presented numbers within a certain range in a bimanual task tend to respond faster to relatively small numbers with a left key and to relatively large numbers with a right key. This effect has been interpreted as evidence that humans mentally represent numbers from left to right according to a mental number line (MNL; Restle, 1970). Several different accounts have then emerged to explain the functioning of the SNARC effect. For example, recently Felisatti et al. (2020) suggested that the mechanism underlying the SNARC effect might be unrelated to numbers but rather be of biological nature, based on the fact that the human brain would respond to different spatial frequencies with an asymmetric tuning.

With the increasing interest in the SNARC effect, it soon became clear that the way in which we spatially map numbers is not fixed and immutable but is rather flexible. For example, the same number can be associated to opposite response sides depending on which stimuli range is considered (Dehaene et al., 1993, Experiment 3; Roth et al. 2025a, b). Our spatial representation of numbers is also deeply influenced by the context in which we encounter them, as well as by task requirements (Mingolo et al., 2021). For instance, the context can alter the SNARC effect if it activates opposite scanning direction in participants that speak different languages with different reading/writing directions (Shaki & Fischer, 2008). Moreover, if the task requires to process numbers in the context of a clockface, in which small/large numbers are represented on the opposite sides compared to the MNL, commonly observed biases such as the SNARC effect or the Small Number Bias can be significantly altered or reversed (Bächtold et al., 1998; Mingolo et al., 2024, 2025). In general, when the context or the task demands are subjected to various situated influences, different alterations of the SNARC effect can emerge (Cipora et al., 2018).

Despite many existing theories accounting for the SNARC effect, the mechanisms underpinning it are still debated. It is still not clear how, exactly, order and magnitude are spatially mapped in numbers. Indeed, a number conveys both information about quantity, defined “magnitude” (e.g., 3 is smaller than 4), and information about order (e.g., the 3rd comes before the 4th ). But in numbers, order and magnitude covary; either due to their order or due to their magnitude small numbers are associated to the left and large numbers are associated to the right. That is, independently from order, small magnitudes would be mapped on the left (Walsh, 2003). Similarly, independently from magnitude, the first numbers would be mapped on the left (van Dijck & Fias, 2011). For this reason, it is very difficult to determine whether small numbers are associated to the left due to their order, or to their magnitude. Converging evidence suggests that magnitude-based spatial associations may be more closely linked to phylogenetically ancient neural mechanisms (Toomarian & Hubbard, 2018); on the other hand, it could be argued that order-based associations likely reflect cultural and experiential learning (but see Rugani et al., 2010 for evidence on the biological-based account for numerical order).

Evidence supporting the role of order for spatial-numerical associations (SNAs) was provided by Gevers et al. (2003; but see also Roth et al. 2025a, b). The study showed the occurrence of SNARC-like effects for letters, an overlearned set of stimuli that do not possess magnitude properties (i.e., left-key advantage for the first letters of the alphabet and right-key advantage for the last). The relevance of a culturally learned order for SNAs is as well demonstrated by various studies based on reading-writing habits (e.g., Shaki et al., 2009). Moreover, the working memory account for SNAs provides a strong argument in favor of order, considering the evidence that newly acquired sequences of numbers are spatially mapped according to their ordinal position in working memory, and not to the MNL (van Dijck & Fias, 2011).

Conversely, the role of magnitude is supported by the observed spatial associations for magnitudes that do not have a culturally overlearned order. For instance, luminance (Fumarola et al., 2014) and animals’ typical size (Sellaro et al., 2015). Moreover, the small-left and large-right associations were found in newborn chicks (Rugani et al., 2015, 2020) and human neonates too (Di Giorgio et al., 2019). This finding suggests that the left-to-right spatial mapping of magnitudes may be based on innate mechanisms, independent of the culturally acquired order. In line with this, from a theoretical perspective, the ATOM (A Theory of Magnitude) model (Bueti & Walsh, 2009; Walsh, 2003) suggests that all quantities are spatially mapped, stating also that this shared magnitude system would originate in early childhood.

Past research suggests, accordingly, that both order and magnitude are relevant for SNARC-like effects (Prpic et al., 2021). Although it is difficult to dissociate their contribution because these two factors naturally confound in numbers. An attempt to disambiguate the roles of order and magnitude was made by Prpic et al. (2016), who tested expert musicians using musical note values. This kind of stimuli was used because they are typically represented in a descending order, starting from the largest value, and progressing to the smaller one. The participants had to perform either a direct task (i.e., a note value comparison) or indirect ones (i.e., a line orientation judgement and a detection task) on pictures representing musical notes. They found that, depending on the task, spatial associations were either in line with the magnitude or with the order of the stimuli. The authors proposed that two separate mechanisms elicit SNARC-like effects: one based on order (Order-Related Mechanism - ORM) and one based on magnitude (Magnitude-Related Mechanism - MRM).

The model proposed by Prpic et al. (2016) states that the two mechanisms are activated depending on the task requirements. The ORM would be mainly activated by direct tasks, which require to directly compare a feature of the stimuli with a reference (e.g., magnitude classification). Indeed, to judge whether a quantity is smaller or larger than a reference, an ordinal comparison between the two would be necessary. On the other hand, the MRM would be mainly activated by indirect tasks, which require participants to judge a feature of the stimuli irrelevant for the study (e.g., orientation judgement). Thus, according to this model, different tasks can unveil the predominance of the different mechanisms underlying SNARC-like effects.

Previous attempts to separately investigate order and magnitude in SNARC-like effects were either done on non-numerical stimuli or based on manipulations that altered the natural order of representation of the stimuli. For instance, van Dijck and Fias (2011) observed the ordinal position effect, namely the spatial mapping of number’s order, by transitorily altering the order of numbers in working memory. Prpic et al. (2016) used non-numerical stimuli (i.e., musical notes values) which are only familiar to the population of musicians and found that the order of notes reversed the SNARC effect only in a direct task. Mingolo et al. (2021) altered the natural order of numbers by making participants process numbers in an atypical spatial numerical configuration and found that the alternative order can alter the SNARC effect only if it is further reinforced by task demands.

Recently, an attempt to disambiguate order and magnitude in numerical stimuli without altering their order has been done by Koch et al. (2023). They manipulated the set of stimuli in a way that could dissociate the contribution of ordinal and magnitude number representations. Namely, the sets they selected consisted in three consecutive numbers and a fourth maximally distant one (e.g., 1–2 – 3–8, where 8 is just one unit apart from 3 in an ordinal perspective and five units apart in a magnitude one). The results of the study are better described by the magnitude model; however, order seems to have played a role as well. Hence, the disambiguation of order and magnitude using numerical stimuli is still an open question.

### The present study

The aim of the present study is to disambiguate the role of order and magnitude in the SNARC effect, without artificially manipulating the order of numbers, and using numerical stimuli familiar to most people. Instead of manipulating the natural order of numbers, the present study uses a novel kind of stimuli that consist in a particular representation of numbers known to most people, namely playing cards, given that they are spatially organized differently among people.

**Fig. 1:**
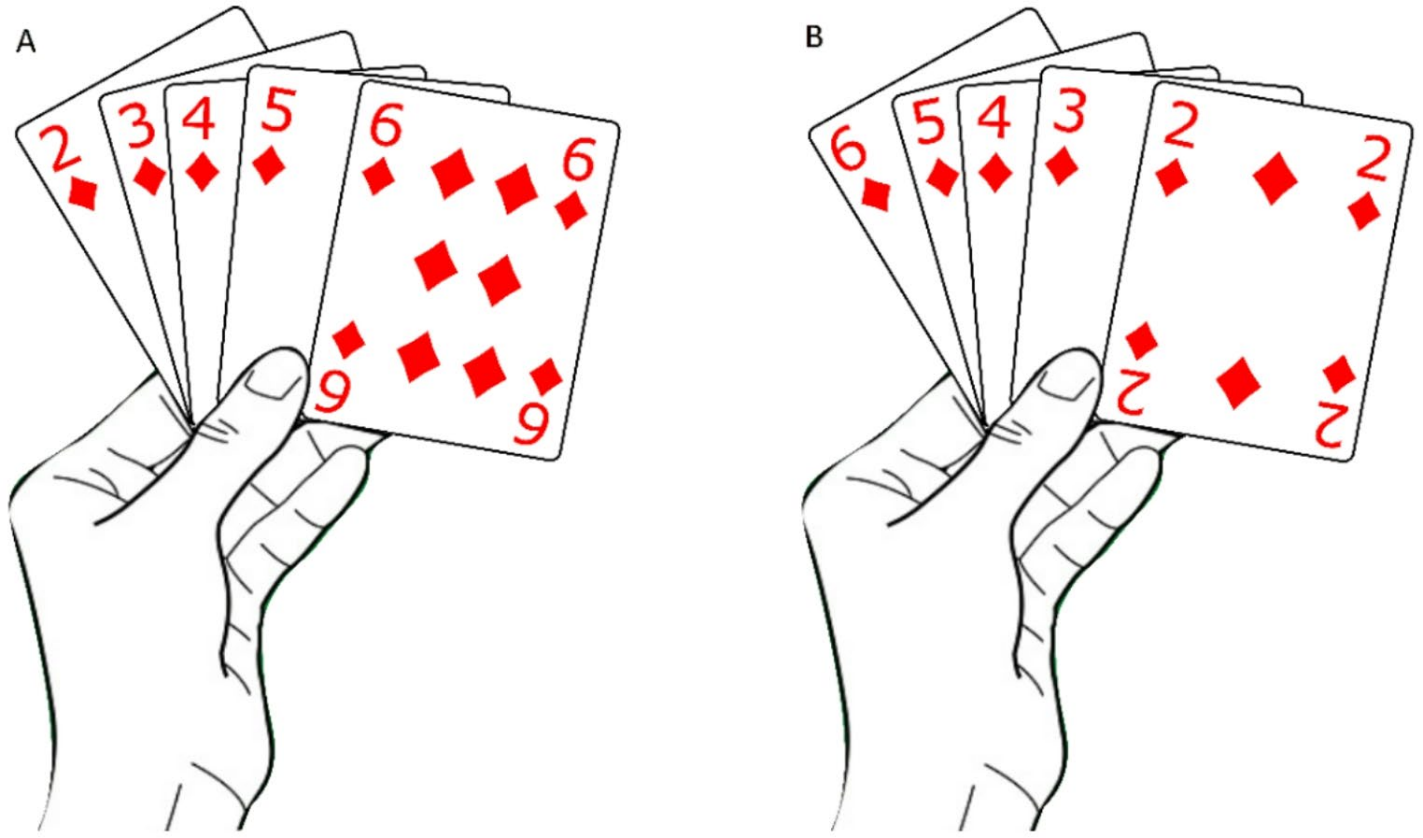
Individual order of the disposition of cards: ascending order - AO (panel **A**) and descending order - DO (panel **B**)

When playing cards, people stably arrange cards according to their individual order of disposition. Most people hold them in *ascending order* (“AO”; Fig. 1A), namely they arrange low value cards to the left and high value cards to the right. This arrangement is consistent with the typical left-to-right mapping of numbers and, in general, with the SNARC effect. For AO individuals, card order and card magnitude would elicit consistent representations, which should reflect a regular, left-to-right, SNARC-like effect for cards.

Conversely, a minority of people (around 15%, according to our data collection experience) spontaneously and systematically hold cards in *descending order* (“DO”; Fig. 1B), namely they arrange high value cards to the left and low value cards to the right. Thus, according to their individual order of disposition, DO people should associate small value cards (e.g., 2) to the right, and large value cards (e.g., 6) to the left. However, according to their intrinsic magnitude, the same cards are expected to be associated with the opposite spatial coordinates: small value cards to the left, and large value cards to the right. Hence, for DO individuals, card order and magnitude should elicit opposite spatial mappings. It is not clear whether this inconsistency would determine a regular SNARC-like effect for cards stimuli, determined by card magnitude, or a reversed SNARC-like effect due to card order.

According to the CORrelations in Experience (CORE) principle (Pitt & Casasanto, 2020), the MNL is shaped by specific experiences that spatialize numbers, such as repeatedly seeing numbers arrayed in a certain way, thus based on order rather than magnitude. In their study, participants were trained to read mirror-reversed text (Experiment 1), or to count on fingers from right to left (Experiment 2 and 3). Only the reversed finger counting training altered the MNL, because among the two it was the only experience that spatializes numbers. Based on the CORE principle, we can hypothesize that a similar effect with cards could emerge. Namely, the specific experience accumulated with playing cards should lead participants to spatialize cards according to the individual order of disposition (i.e., in ascending vs. descending order). Therefore, the CORE principle would predict that order would prevail on magnitude, determining a reversed SNARC-like effect when DO participants classify card values. However, it is reasonable to expect that this specific experience would have an effect only within the domain of cards and would not extend to that of numbers in general, given the large amount of experience with the left-to-right mapping of numbers in Western cultures. Consequently, order-based effects with cards may emerge only when the card-specific ordering is sufficiently salient to override the highly overlearned number-based mapping.

In the present study, we conducted three experiments testing both DO and AO participants. The study employs two direct tasks, using cards or numbers as stimuli. According to Prpic et al. (2016), the use of direct tasks would induce the processing of the ordinal properties of the stimuli. According to this hypothesis DO participants – who have opposite mappings of card order and magnitude – should exhibit the regular SNARC effect with numbers, and a reversed SNARC-like effect with cards. Conversely, in the same task with cards, AO participants should exhibit a regular SNARC-like effect.

Importantly, these effects are expected to occur at group level and not at individual level, since the SNARC effect is not always stable and consistent across individuals (Cipora et al., 2019; Wood et al., 2006). Despite this individual instability, we expect that both AO and DO participants will show a regular SNARC effect with numbers, since they belong to a Western culture and are used to the left-to-right representation of regular numbers. Conversely, cards would spontaneously elicit an overlearned order (descending or ascending) that is context-specific and limited to that category of stimuli. Using this type of stimuli, it will be possible to disentangle the contribution of order and magnitude without “artificially” altering the order of numbers through experimental manipulations.

## Experiment 1

The first experiment was performed by a sample of participants belonging to the DO category. The same participants performed a magnitude classification task on numerical stimuli in Experiment 1a and on cards stimuli in Experiment 1b. In Experiment 1a we expected to observe a regular SNARC effect, since these participants should have a left-to-right representation of numbers consistently elicited by both number’s order and magnitude. In Experiment 1b different outcomes could emerge, depending on which mechanism between order and magnitude prevails, given that they would elicit opposite mappings in these participants. According to the prediction made by Prpic et al.’s model (2016) on direct tasks, participants should exhibit a reversed, right-to-left SNARC, determined by the predominance of order. On the other hand, if DO participants exhibit a regular left-to-right SNARC, this would indicate the predominance of magnitude.

### Method

#### Participants

We tested 56 students from the University of Trieste (50 females, 6 males) belonging to the DO category, with a mean age of 21.00 years (SD = 3.05). Sample size calculation was performed with G*Power using the following parameters for two-tailed one-sample *t* test: power = 0.95, α = 0.05, Cohen’s *d* = 0.50 (medium effect size, in line with previous studies on context manipulation in the SNARC effect: Mingolo et al., 2021; Bächtold et al., 1998); the outcome was a suggested sample size of 54 participants. The sample size of 56 also gives power of 0.80 to detect smaller effect sized (*d* = 0.38). Moreover, we designed the experiments to have 20 repetitions per stimulus and recruited a number of participants considered “very large”, according to the guidelines provided by Cipora and Wood (2017).

All participants had normal or corrected-to-normal vision and one participant was left-handed; all of them were used to the left-to-right reading/writing direction. Furthermore, we ascertained that their psychophysiological state was not affected by alcohol consumption or insufficient sleep in the 24 h preceding the experiment (Murgia et al., 2020). Participants provided written informed consent before taking part to the experiment; the experiment was conducted in accordance with the ethical standards established by the Declaration of Helsinki and with the agreement of the University of Trieste Ethics Committee.

#### Apparatus and stimuli

The experiment was designed and run through the open-source software Psychopy (Peirce et al., 2019), in the version 3.0. The computer used to control the experiment was a Dell desk computer with Intel Core i5 (RAM: 4 Gb). Instructions and stimuli were displayed on a Quato Intelli Proof 242 excellence (24 inches) monitor, with a 1024 × 768 resolution. Participants’ responses were collected through a five-button serial response box.

**Fig. 2:**
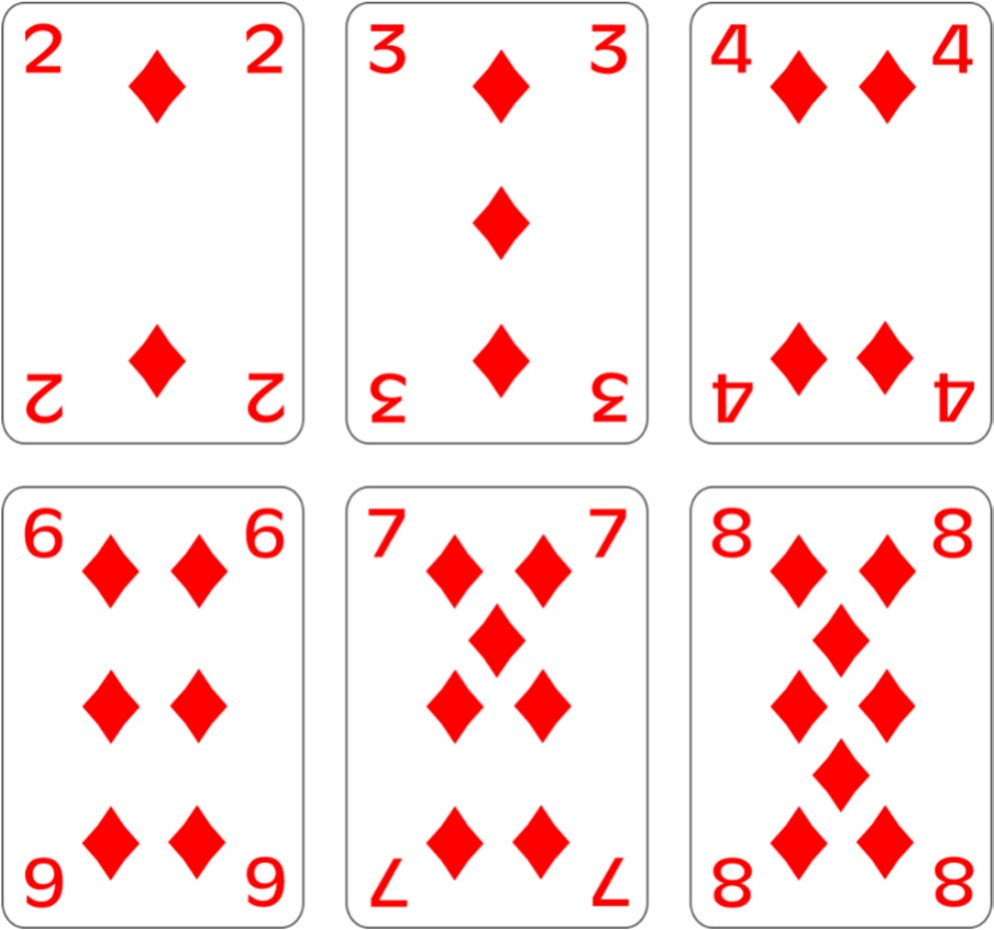
Cards pictures presented as stimuli in card value classification task (Experiment 1b). The reference value was 5, and a picture of the card with the value of 5 was presented at the beginning of the experiment, during instruction presentation

The stimuli displayed in Experiment 1a (magnitude classification) consisted in single digit numbers, presented at the center of the screen, in white against a grey background. The stimuli set was the following: 2–3 – 4–6 – 7–8. The stimuli displayed in Experiment 1b (card value classification) consisted in pictures of playing cards (Fig. 2), presented at the center of the screen, in color. The set included cards with the same values as the numbers used in Experiment 1a, namely: “Two of diamonds”, “Three of diamonds”, “Four of diamonds”, “Six of diamonds”, “Seven of diamonds” and “Eight of diamonds”. The cards presented were all the same suit to avoid effects driven by cards color or suit.

#### Procedure

##### a. Assessment of participants’ individual order of disposition

A crucial passage of the present study was the assessment of participants’ individual order of disposition of cards, which allowed to identify whether a participant arranged cards in ascending or descending order. This procedure had to be as neutral and ecological as possible, to observe the participants’ instinctive behaviour without influencing it, and to be confident about the stability of their behaviour in time. For these reasons, we articulated the assessment in two parts, which occurred in separate occasions, and we designed the assessment as an ecological card game simulation.

The first part of the assessment took place before the beginning of the first experiment, which, due to counterbalancing, could be either Experiment 1a (magnitude classification) or Experiment 1b (card value classification). To avoid influences of the screening on participants’ performance in the experiments, the screening took place a few days before the first experiment. During the card game simulation, the participant was invited to sit at a desk, on which 6 shuffled cards were placed face down. The cards were the same as those displayed as stimuli in Experiment 1b (Fig. 2). The experimenter gave the same instruction to every participant, which exact wording was: “Please, pick up the cards and arrange them in your hands as you were about to start playing a card game. When you are satisfied with the arrangement, put the cards down, face up.” When the participant put down the cards, the experimenter simply took note of the arrangement exhibited by the participant (namely ascending or descending order) without giving any further information.

The second part of the assessment took place at the end of the second experiment. In this case, participants were once again asked to arrange the same cards as if they were about to start playing a card game, and once again the experimenter took note of which arrangement was exhibited. Furthermore, for the final step of the assessment (which concluded the experiments as well), participants filled in a questionnaire. The questionnaire presented two pictures displaying the two possible card arrangements (ascending and descending order). Participants were asked to indicate, on a scale from 1 to 10, how likely it was for them to arrange cards in each order (1 = “very unlikely”, 10 = “very likely”). Moreover, participants were asked to report how they usually represented the order of numbers. Answer options were: “I normally represent numbers in ascending order (e.g., 1-2-3-4-5-6-7-8-9)” or “I normally represent numbers in descending order (e.g., 9-8-7-6-5-4-3-2-1)”.

Depending on the arrangement they exhibited, and on the matching between the information obtained in the first and second part of the assessment, participants were either included in the AO or in the DO group. Participants who exhibited different arrangements in the two parts of the screening, or who gave uncertain answers to the questionnaire (namely scores between 4 and 6 for both arrangements), could not be included in any group.

Finally, the questionnaire also included two questions aimed at investigating participants’ habits regarding card games. The first question asked how frequently participants played cards in everyday life. The possible answers ranged on a scale from 0 to 4: 0 = “never”, 1 = “less than once per month”, 2 = “once or twice per month”, 3 = “once a week”, 4 = “more than once a week”. The second question asked which was their level of expertise with card games on a scale from 0 to 5: 0 = “very low”, 1 = “low”, 2 = “average”, 3 = “high”, 4 = “very high”, 5 = “professional”.

##### b. Tasks

The experiment took place in a quiet room, using dim lights. Participants each sat on a chair at a viewing distance of approximately 60 cm from the PC screen, with their bodies aligned to the midline of the screen. They had a response box in front of them and were instructed to put their left index finger on the leftmost key and their right index finger on the rightmost key.

In Experiment 1a each participant performed a magnitude classification task, which was divided into two blocks (block A and block B). Each block included a practice session, composed of 30 trials, which was not considered for data analysis. During the practice session, trials started with a fixation cross at the centre of the screen, which lasted for 500 ms and was followed by an interstimulus interval (ISI) of 500 ms. After the ISI, the target stimulus (a single-digit number) appeared at the centre of the screen, and lasted until a response occurred, within a response time deadline of 2000 ms. Participants responded by pressing the leftmost or the rightmost key of the response box and, specifically for practice purposes, received feedback about their accuracy (“Correct!” or “Wrong!”) and RT was displayed.

After practice session, each block presented an experimental session, composed by 120 trials. In the experimental session, the structure of the trials was the same as in practice session, as well as the trials’ timings except for the fact that no feedback was given. In Experiment 1a, the instructions of the magnitude classification task required participants to “report whether the presented number was smaller or larger than 5” by pressing the leftmost or the rightmost key of the response box. Depending on the block, the combination of the response buttons could be either SNARC-congruent (“left = smaller” / “right = larger”) or SNARC-incongruent (“left = larger” / “right = smaller”). The combination of response buttons was reversed from block A to block B, and the order of presentation of the two blocks was counterbalanced among participants. If needed, participants were allowed to take a short break between the two blocks, otherwise they continued with the experiment. Instructions explicitly asked participants to be as accurate and as fast as possible.

In Experiment 1b participants performed a card value classification task. The procedure was exactly the same as in Experiment 1a, but this time participants judged cards stimuli instead of numbers. The instructions required participants to “report whether the value of the presented card was smaller or larger than 5”, by pressing the leftmost or the rightmost key of the response box, depending on the response button combination. The two experiments were performed in separate days (with an interval of seven days on average) to prevent any effects of one task on the other. Furthermore, the order of administration of the two tasks was counterbalanced with half of participants in each experiment completing the cards task first and half completing the numbers task first.

### Data analysis, results, and discussion

The independent variables were Hand (left vs. right) and Stimulus (2–3 – 4–6 – 7–8), the dependent variable was the Response Time (RT). RTs of incorrect trials were not included in data analysis. Similarly, RTs shorter than 150 ms or those that differed by more than 2.5 standard deviations from a participant’s mean RT were considered outliers and removed from data analysis. Mean RTs of the correct trials for the left and right hand were computed separately for each participant for each number. To obtain the dRTs, the mean RTs of the left hand were subtracted from the mean RTs of the right hand: dRT = RT (right hand) – RT (left hand). Positive dRTs indicate faster responses with the left hand, whereas negative dRTs indicate faster responses with the right hand.

To determine if the SNARC effect emerged, a regression analysis was conducted (Fias et al., 1996; Lorch & Myers, 1990). A regression equation was computed for each participant with the variable Stimulus as predictor, and dRTs as dependent variable. A two-tailed one-sample *t* test was performed on the regression weights of all equations to test whether they significantly deviated from zero at the sample level.

To examine whether individual differences in card playing experience were associated with the strength of spatial-numerical associations, we conducted correlation analyses between participants’ self-reported card playing frequency and expertise and their individual SNARC slopes for both card and number stimuli, as well as with the difference between them (cards slopes – number slopes) for both frequency and expertise.

Finally, we checked if task order affected participants performance through an independent sample *t* test comparing participants who completed the card task first versus those who completed the number task first. Notably, no significant effect emerged in any of the three experiments; for this reason, these results are not reported in the result sections of the next experiments.

In Experiment 1a, the average RT was 374.32 ms (SD = 52.17 ms), the overall proportion of errors was 7.48%, and the amount of outlier RTs discarded from data analysis was 2.36%. In Experiment 1b, the average RT was 390.22 ms (SD = 62.17 ms), the overall proportion of errors was 8.88%, and the amount of outlier RTs discarded was 2.86%. As for the frequency and expertise with card games, on average participants reported playing cards once or twice a month (M = 2.07, SD = 0.94) and to have an average level of expertise (M = 2.26; SD = 1.01).

**Fig. 3:**
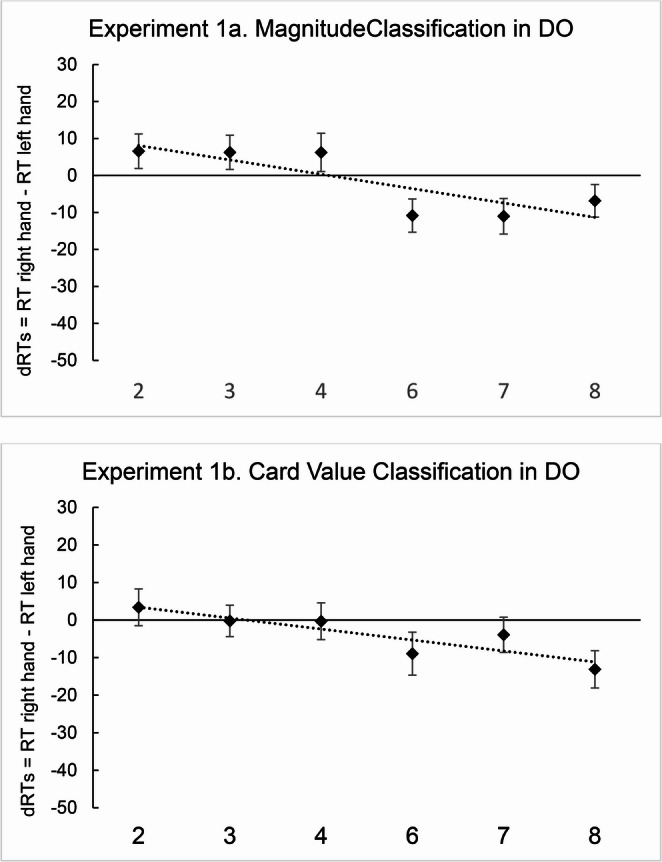
Mean dRTs (right key - left key) for every numerical stimulus in the magnitude classification (Experiment **1a**) and in the card value classification task (Experiment **1b**) in DO participants. Positive differences indicate faster left-key responses; negative differences indicate faster right-key responses. Error bars indicate the standard error of the mean

In Experiment 1a, the results showed that the regression weights deviated significantly from zero [*M*_*slopes*_ = -15.81; *t*(56) = -2.26; *p* = .028; *d* = -0.29], consistently with the SNARC effect (Fig. 3, Experiment 1a). Differently, in Experiment 1b, the one-sample *t* test showed that the regression weights did not deviate significantly from zero [*M*_*slopes*_ = -9.61; *t*(56) = -1.25; *p* = .218; *d* = -0.16] (Fig. 3, Experiment 1b). A two-tailed paired sample *t* test showed that there was no difference between the slopes in Experiment 1a and 1b [*M*_*1a*_ = -15.81, *M*_*1b*_ = -9.61; *t*(56) = -0.61; *p* = .541; *d* = -0.08].

For card stimuli (Experiment 1b), significant positive correlations emerged between card playing frequency and SNARC slope (*r* = .266, *p* = .046) and between expertise and SNARC slope (*r* = .279, *p* = .035) for card stimuli. These positive correlations indicate that greater card playing experience was associated with less negative SNARC slopes, in line with a weaker standard left-to-right spatial-numerical association. Notably, no significant correlations were found between card playing experience and SNARC slopes for number stimuli (Experiment 1a; frequency: *r* = − .201, *p* = .135; expertise: *r* = − .241, *p* = .071), suggesting that the modulation effect was specific to card stimuli rather than reflecting a general influence on numerical processing. Interestingly, an exploratory analysis examined whether card playing experience was associated with the difference between card and number SNARC slopes (calculated as cards slopes – numbers slopes). Interestingly, significant positive correlations emerged between this difference score and both frequency (*r* = .388, *p* = .003) and expertise (*r* = .394, *p* = .002), indicating that greater card playing experience was associated with larger discrepancies in how cards versus numbers were spatially mapped.

DO participants exhibited the SNARC effect only in the magnitude classification task performed on numbers, while the same effect did not emerge in the card value classification task. However, the direct comparison between the two did not indicate significant differences. Results from Experiment 1b, thus, did not give a clear indication about the predominance of order or magnitude. This could indicate that order was not the only mechanism involved in card processing, with magnitude having a certain influence as well. Moreover, we cannot exclude that the lack of SNARC effect was determined by the particular kind of stimuli used. To rule out the possibility that these stimuli prevented the SNARC effect from emerging, in Experiment 2 we ran the magnitude classification task and card value classification task on a sample of AO participants.

## Experiment 2

In Experiment 1, we observed that DO participants do not exhibit the SNARC effect in the card value classification task. However, we do not know whether the effect failed to emerge because of the particular kind of stimuli we used. In Experiment 2, a sample of participants belonging to the AO category – who have consistent mappings of card order and magnitude – performed a magnitude classification task on numerical stimuli in Experiment 2a and on cards stimuli in Experiment 2b. In this way, we could test whether participants in which cards’ order and magnitude elicit the same mapping exhibit a regular SNARC effect when they classify numbers and cards stimuli.

### Method

#### Participants

We tested 71 students from the University of Trieste (61 females, 10 males) belonging to the AO category, with a mean age of 20.83 years (SD = 1.32). The sample size was determined with the same method used in Experiment 1, which indicated 54 participants to be the minimum required sample size. We exceeded this threshold to be conservative in our approach to statistical power. All participants had normal or corrected-to-normal vision and one participant was left-handed; all of them were used to the left-to-right writing direction. Furthermore, we ascertained that their psychophysiological state was not affected by alcohol consumption or insufficient sleep in the 24 h preceding the experiment (Murgia et al., 2020). Participants provided written informed consent before taking part to the experiment; the experiment was conducted in accordance with the ethical standards established by the Declaration of Helsinki and with the agreement of the University of Trieste Ethics Committee.

#### Apparatus and stimuli

The apparatus employed in Experiment 2 was the same as in Experiment 1.

#### Procedure

The assessment of the individual order of disposition was performed as previously described and the procedure of the experiment was the same as in Experiment 1.

### Data analysis, results, and discussion

In Experiment 2a, the average RT was 380.07 ms (SD = 55.33 ms), the overall proportion of errors was 5.58%, and the amount of outlier RTs discarded from data analysis was 2.11%. In Experiment 2b, the average RT was 404.32 ms (SD = 64.40 ms), the overall proportion of errors was 5.22%, and the amount of outlier RTs discarded was 2.64%. As for the frequency and expertise with card games, on average participants reported playing cards between less than once a month and once/twice a month (M = 1.62, SD = 1.01) and that their level of expertise ranges between low and average (M = 1.73; SD = 1.05).

**Fig. 4:**
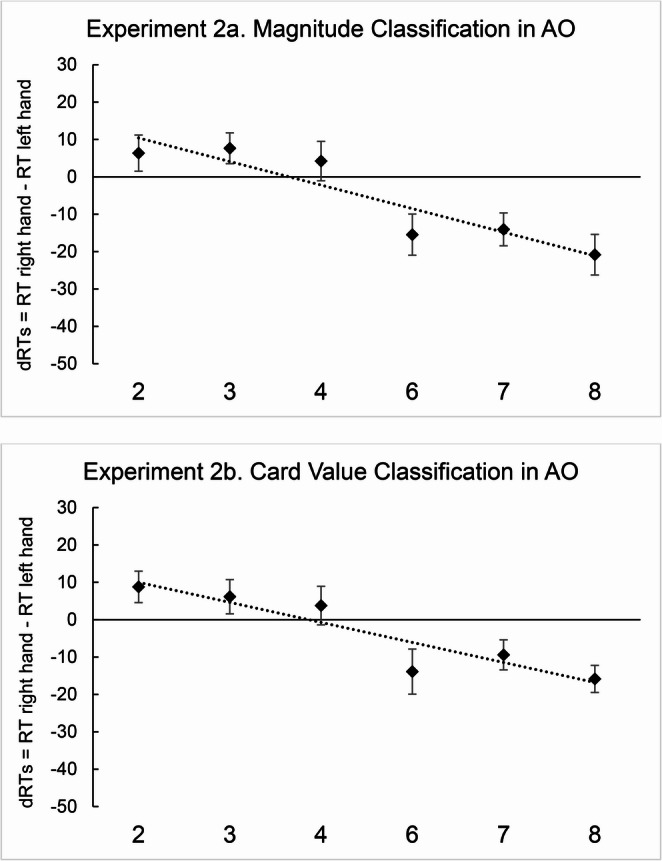
Mean dRTs (right key - left key) for every numerical stimulus in the magnitude classification (Experiment **2a**) and in the card value classification task (Experiment **2b**) in AO participants. Positive differences indicate faster left-key responses; negative differences indicate faster right-key responses. Error bars indicate the standard error of the mean

In Experiment 2a, the two-tailed one-sample *t* test showed that the regression weights deviated significantly from zero [*M*_*slopes*_ = -22.82; *t*(70) = -2.86; *p* = .006; *d* = -0.33], in the direction of the SNARC effect (Fig. 4, Experiment 2a). Similarly, in Experiment 2b, the regression weights deviated significantly from zero [*M*_*slopes*_ = -19.37; *t*(70) = -2.70; *p* = .009; *d* = -0.32], consistently with the SNARC effect (Fig. 4, Experiment 2b). A two-tailed paired sample *t* test showed that there was no difference between the slopes in Experiment 2a and 2b [*M*_*2a*_ = -22.82, *M*_*2b*_ = -19.37; *t*(70) = -0.41; *p* = .684; *d* = -0.04].

No significant correlations emerged between card playing experience (frequency or expertise) and SNARC slopes for either card stimuli (Experiment 2b; frequency: *r* = .055, *p* = .651; expertise: *r* = − .033, *p* = .783) or number stimuli (Experiment 2a; frequency: *r* = .097, *p* = .423; expertise: *r* = .124, *p* = .302). Finally, no significant positive correlations emerged between card playing frequency and the difference between cards and numbers SNARC slopes for both frequency (*r* = − .129, *p* = .249) and expertise (*r* = − .126, *p* = .294). This null result is consistent with the alignment between card and number ordering in AO participants, where both mappings follow the same left-to-right direction. Overall, this pattern of results contrasts with the findings in DO participants (Experiment 1), suggesting that the relationship between card playing experience and spatial-numerical mappings may be specific to individuals whose card arrangement practices conflict with the culturally dominant left-to-right ordering.

Results suggest that AO participants exhibited a regular SNARC effect both in the magnitude classification task and in the card value classification task. For this reason, we can rule out the possibility that card stimuli prevented the SNARC effect from emerging and we can affirm that cards are a suitable stimulus to investigate the SNARC effect. To further clarify the ambiguous result observed in Experiment 1b, we performed an online replication on DO participants in Experiment 3.

## Experiment 3

Experiment 1b found that DO participants do not show the SNARC effect in the card value classification task. In Experiment 2 it was ascertained that card stimuli do not prevent the SNARC effect to occur. However, we still do not know whether the lack of the SNARC effect observed in Experiment 1b was due to the conflicting representations elicited by order and magnitude or to the fact that there could still have been a certain variability in the sample.

Experiment 3 consisted in a replication of Experiment 1 conducted online, allowing the easier recruitment of a large sample of DO participants. The same participants performed a magnitude classification task in Experiment 3a and a card value classification task in Experiment 3b. As in Experiment 1, two possible outcomes could emerge. If order prevails on magnitude, a reversed SNARC effect should emerge in Experiment 3b. If magnitude prevails on order, a regular SNARC effect should emerge.

### Method

#### Participants

Seventy participants belonging to the DO category were recruited via the platform Prolific; 66 of them completed all parts of the experiment (39 females, 27 males) with a mean age of 28.21 (SD = 4.20); nine participants were left-handed. The sample size was determined with the same method used in Experiments 1 and 2 which indicated 54 participants to be the minimum required sample size. We exceeded this threshold to be conservative in our approach to statistical power. Participants provided informed consent before taking part to the experiment; the experiment was conducted in accordance with the ethical standards established by the Declaration of Helsinki and with the agreement of the University of Trieste Ethics Committee.

#### Apparatus and stimuli

The experiment was designed on Pavlovia and Qualtrics and conducted online, so the apparatus was replaced by participants’ own laptops. Responses were given through the keyboard using the “a” and “l” keys described in Experiment 1. Stimuli were the same as in Experiment 1.

#### Procedure

##### a. Assessment of participants’ individual order of disposition

As in the experiments conducted in person, participants’ individual order of disposition of cards was carried out before the beginning of Experiment 3. The screening was conducted on an initial sample of 800 participants. The procedure was articulated in a two-parts survey, conducted in Qualtrics. The first part of the assessment took place a few days before the beginning of the first experiment and consisted of a questionnaire. The first question presented two pictures exemplifying the two possible arrangements (i.e., ascending, and descending order, see Fig. 1), and asked participants “When you play cards, in which of these two arrangements do you typically arrange them in your hands?”. Response options were “ascending order”, “descending order” or “I don’t know”. Then, participants were asked to rate how likely it was for them to arrange cards in either arrangement on a scale from 1 to 10 (1 = very unlikely, 10 = very likely).

The second and final part of the assessment took place at the end of the second experiment and consisted in another questionnaire. In this case, participants were once again asked to report what was their usual card arrangement. Moreover, participants were asked to report how they usually represented number’s order. Answer options were: “I normally represent numbers in ascending order (e.g., 1-2-3-4-5-6-7-8-9)” or “I normally represent numbers in descending order (e.g., 9-8-7-6-5-4-3-2-1)”.

Participants who selected the options “ascending order” or “I don’t know” when asked about their favourite card arrangement in the initial screening survey or who gave uncertain answers (namely scores between 4 and 6 for both arrangements) could not be recruited and were compensated for the screening survey. Participants who selected the option “descending order” (*n* = 105) were invited to perform the experiment, and 66 of them completed it. Among the participants who completed the experiment, 7 reported to prefer the ascending arrangement in the second part of the screening and were therefore excluded from data analysis. All participants reported to represent numbers in ascending order. As in the previous two experiments, in the final questionnaire participants were asked how frequently they played cards in everyday life on a scale from 0 to 4 and which was their level of expertise with card games on a scale from 0 to 5.

##### b. Tasks

The experiment was performed online on the platform Pavlovia. In Experiment 3a participants performed a magnitude classification task, and in Experiment 3b they performed a card value classification. The structure and the instructions of the experiment were exactly the same as in Experiment 1.

### Data analysis, results, and discussion

In Experiment 3a, the average RT was 481.18 ms (SD = 94.24 ms), the overall proportion of errors was 7.05%, and the amount of outlier RTs discarded from data analysis was 2.68%. In Experiment 3b, the average RT was 493.16 ms (SD = 92.25 ms), the overall proportion of errors was 4.77%, and the amount of outlier RTs discarded was 3.04%. As for the frequency and expertise with card games, on average participants reported playing cards between less than once a month and once/twice a month (M = 1.59, SD = 1.26) and that their level of expertise ranges between low and average (M = 1.45; SD = 1.02).

**Fig. 5:**
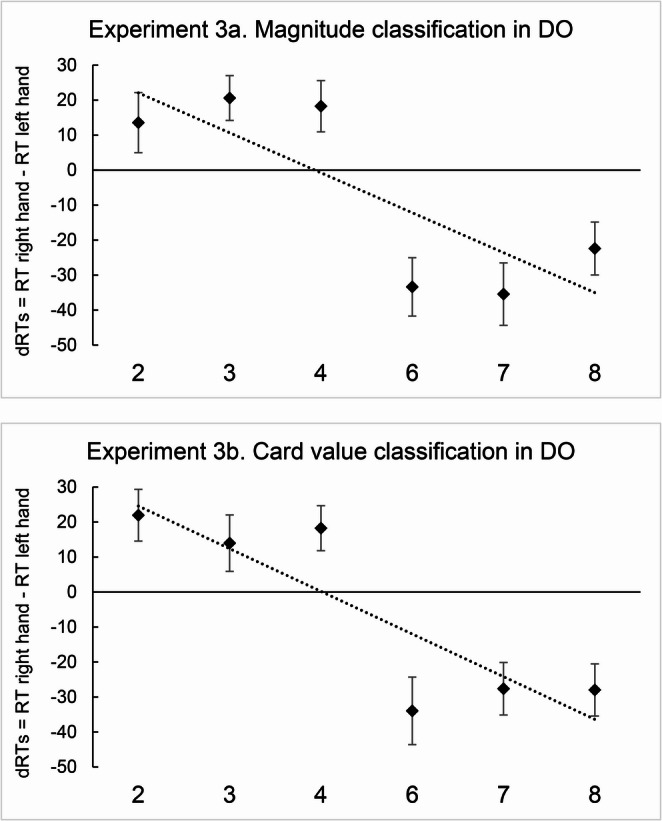
Mean dRTs (right key - left key) for every numerical stimulus in the magnitude classification (Experiment **3a**) and in the card value classification task (Experiment **3b**) in DO participants. Positive differences indicate faster left-key responses; negative differences indicate faster right-key responses. Error bars indicate the standard error of the mean

In Experiment 3a the two-tailed one-sample *t* test conducted on regression weights suggested that they deviated significantly from zero [*M*_slope_ = -47.88; *t*(58) = -3.43; *p* = .001; *d* = -0.44], consistently with the SNARC effect (Fig. 5, Experiment 3a). Similarly, in Experiment 3b the results showed that the regression weights deviated significantly from zero [*M*_slope_ = -47.91; *t*(58) = -3.92; *p* < .001; *d* = − 0.51], in the direction of the SNARC effect (Fig. 5, Experiment 3b). A two-tailed paired sample *t* test showed that there was no difference between the slopes in Experiment 3a and 3b [*M*_*3a*_ = -47.9 ms/digit, *M*_*3b*_ = -47.9; *t*(58) = 0.001; *p* = .99; *d* = 0.00].

Correlation analyses revealed a significant positive relationship between card playing frequency and SNARC slope for card stimuli (*r* = .267, *p* = .041), with greater frequency associated with weaker standard SNARC effects. Instead, the correlation with expertise was not significant (*r* = .068, *p* = .608). As in Experiment 1, no significant correlations were found between card playing experience and SNARC slopes for number stimuli (frequency: *r* = .097, *p* = .466; expertise: *r* = − .235, *p* = .074), further supporting the stimulus-specific nature of this modulation effect. Finally, no significant positive correlations emerged between card playing frequency and the difference between cards and numbers SNARC slopes for both frequency (*r* = .054, *p* = .648) and expertise (*r* = .230, *p* = .080). Here, the lack of significant correlations, despite a trend for expertise, likely reflects the lower average card playing experience in this sample compared to Experiment 1, which may have attenuated the card-number differentiation effect.

Finally, we conducted direct comparisons between the Experiment 1 and Experiment 3 DO samples on card playing experience using Mann-Whitney U tests. For self-reported expertise, a significant difference emerged (*U* = 1072, *p* < .001), with Experiment 1 participants (*M* = 2.16) reporting significantly higher expertise than Experiment 3 participants (*M* = 1.46). For card playing frequency, the difference approached significance (*U* = 1357, *p* = .055), with Experiment 1 participants (*M* = 2.09) showing higher frequency than Experiment 3 participants (*M* = 1.46).

In Experiment 3, DO participants exhibited a regular SNARC effect both in magnitude classification task and in card value classification task. These results did not confirm the finding from Experiment 1, where a null result emerged in card value classification. In this case, results clearly indicate that DO participants show a regular SNARC effect when judging cards. For this reason, it is reasonable to interpret this result as evidence that the SNARC effect was determined by magnitude representation rather than by order. The comparison between the experience of DO participants in Experiment 1 and 3 provides a coherent explanation for the discrepant findings between experiments. In Experiment 1, the higher average card playing expertise probably created greater competition between participants individually practiced right-to-left card ordering and the culturally dominant left-to-right number mapping. This competition, reflected in the positive correlations between expertise and SNARC slopes, attenuated the standard SNARC effect at the group level, preventing it from reaching statistical significance with the available sample size. In Experiment 3, participants had lower average expertise, meaning their card-specific ordering exerted less influence on spatial-numerical associations, allowing the regular SNARC effect to emerge significantly.

## General discussion

The aim of the present study was to disambiguate the role of order and magnitude in the SNARC effect, without artificially manipulating the order of numbers, and using numerical stimuli familiar to most people. To achieve this goal, the study tested participants that arrange playing cards in opposite ways (ascending vs. descending order) in direct tasks performed either on cards or on numbers. In Experiment 1 DO participants showed a regular SNARC effect in magnitude classification, while they exhibited no SNARC effect in card value classification. The different results observed in the two tasks could indicate that DO participants had a different spatial representation of numbers and cards. These results seemed to be influenced by stimuli’s order, since they led to a clear SNARC effect for numbers and to the lack of such effect for cards. However, no inversion of the SNARC effect was clearly observed in card value classification, and thus the influence of magnitude could not be ruled out completely. In Experiment 2, we tested AO participants in magnitude classification and in card value classification to ascertain whether they would exhibit regular SNARC effects. Moreover, we aimed to determine whether the lack of SNARC effect found in Experiment 1b (card value classification in DO participants) could have been determined by cards themselves. Results showed that this was not the case, since AO participants showed a regular SNARC effect with both kinds of stimuli. This experiment confirmed that cards can elicit a SNARC effect. Still, it was not clear whether the lack of SNARC effect in DO reflects the influence of cards’ order or magnitude.

In Experiment 3, the procedure used in Experiment 1 was replicated online. This time results revealed that DO participant exhibited the SNARC effect both in magnitude classification and in card value classification, differently from what was observed in Experiment 1.

The correlation analyses examining the relationship between card playing experience and SNARC slopes provide critical insights into the boundary conditions under which experience-dependent spatial-numerical mappings emerge. A consistent and theoretically informative pattern emerged across experiments: in DO participants (Experiments 1 and 3), greater card playing experience was associated with weaker standard SNARC effects specifically for card stimuli, while no such relationship was observed in AO participants (Experiment 2) or for number stimuli in any group.

Notably, exploratory analyses in Experiment 1 revealed that card playing experience was associated not only with weaker SNARC effects for cards, but also with greater divergence between how cards and numbers were spatially represented. The difference between card and number SNARC slopes showed even stronger correlations with card playing experience, indicating that accumulated experience with card-specific ordering creates increasingly differentiated spatial representations: individuals with greater card playing experience showed substantially weaker SNARC effects for cards while maintaining standard SNARC effects for numbers, resulting in larger discrepancies between the two stimulus types. Rather than producing a general recalibration of spatial- numerical mappings, card playing experience appears to create a context-dependent representational system that is selectively engaged when processing cards but does not extend to numbers presented in isolation.

These findings have several important theoretical implications. First, the positive correlations between card playing experience and SNARC slopes in DO participants suggest that as card-specific experience accumulates, the right-to-left card ordering exerts greater influence on spatial-numerical associations, attenuating – though not reversing – the standard left-to-right mapping. The attenuation rather than reversal likely reflects the competition between two conflicting spatial representations: the highly overlearned left-to-right number sequence and the individually practiced right-to-left card arrangement.

Second, the stimulus-specificity of this modulation – observed exclusively for card stimuli and not for number stimuli – indicates that the experiential effect operates within a circumscribed domain rather than affecting numerical processing globally. This specificity aligns with our prediction that card-specific ordering would be limited to the card domain and would not extend to numbers in general, given the ubiquity and strength of the left-to-right numerical mapping in Western cultures. The robust differentiation effects observed in Experiment 1 provide particularly strong support for this domain-bounded influence of experience.

Third, the absence of correlations in AO participants (Experiment 2) reveals a crucial asymmetry in how experience shapes spatial-numerical associations: when individuals’ card arrangement practices align with the culturally dominant left-to-right direction, increased experience does not modulate the SNARC effect because both sources of spatial-numerical mapping reinforce the same directional association. In contrast, when card arrangement practices conflict with cultural conventions (as in DO participants), experience becomes a critical modulating factor precisely because it strengthens a competing spatial representation. This asymmetry demonstrates that the influence of domain-specific experience on spatial-numerical associations depends critically on whether that experience creates conflict or convergence with broader cultural-numerical practices.

Our results provide important insights into the boundary conditions of the CORE principle (Pitt & Casasanto, 2020). According to CORE, “the way a source and target domain are mapped in the mind is determined by the way those domains are correlated in experience” (p. 1051). Based on this principle, we predicted that DO participants’ specific spatial experience with cards might shape their spatial mapping of card values. However, our results suggest that this prediction requires important refinement. Specifically, while DO participants consistently organize cards from right to left when playing – a behaviour that itself appears contrary to typical Western left-to-right cultural experience – this card-specific ordering did not override the standard left-to-right magnitude mapping when judging card values. Notably, all DO participants reported representing numbers consistently from left to right, indicating they have fully internalized the culturally dominant numerical mapping. The lifelong experience with the left-to-right number sequence in Western cultures far exceeds the more limited, context-specific experience of arranging playing cards. Our results indicate that when these two sources of experience conflict, the more robust and overlearned mapping (numbers) dominates over the more restricted one (cards), even when stimuli are presented in the domain-specific context (playing cards). These findings suggest that the CORE principle may be modulated by the relative strength and frequency of different experiential sources. Rather than contradicting the CORE principle, our findings reveal that its predictions depend critically on the balance between competing experiential sources. The principle may apply most straightforwardly when a single, coherent source of experience dominates, but when multiple conflicting experiential mappings exist, particularly when they differ substantially in strength and frequency, the more robust mapping seems to prevail. This is in line with past studies on the role of spatial-numerical context on the SNARC effect (Mingolo et al., 2021, 2024, 2025). Future research examining individuals with more extensive and frequent card-playing experience or using paradigms that increase the salience of the card-specific context, would provide further tests of these boundary conditions.

Altogether, results suggest a prevalence of magnitude representation over order. This is apparently in contrast with the model described by Prpic et al. (2016), which states that direct tasks preferentially activate the Order Related Mechanism (see also Prpic et al., 2021). However, it is in line with Koch et al. (2023), who found that magnitude explained their data better than order. This finding is supported by ATOM (A Theory of Magnitude; Walsh, 2003). In fact, this theory states that magnitudes belonging to the domains of numbers, time and space are mentally represented in a *generalized magnitude system*. Such a generalized system would explain evidence of SNAs exhibited using different paradigms (Shaki & Fisher, 2014; Sellaro et al., 2015), and different categories of participants, such as human neonates (Di Giorgio et al., 2019) and animals (e.g., 3-days-old chicks; Rugani et al., 2015), who are biased by the left-to-right order preference typical of Western cultures.

The differing trends observed in DO participants between the individual order-of-disposition assessment and the card value classification task may be explained by the distinct nature of these two tasks. These findings support the notion that explicit ordering tasks – such as the one used in our initial assessment – elicit specific types of SNAs that are largely shaped by cultural influences and habitual number-space associations encountered in daily life. Nonetheless, more implicit tasks that do not require explicit ordering of stimuli, like card value classification, appear to elicit different forms of SNAs that are more closely tied to innate biological mechanisms (Eccher et al., 2025).

Furthermore, our results suggest that spatial associations for stimuli stored in long-term memory are different from those arising for stimuli temporarily stored in working memory. As previously discussed, cards are a set of stimuli spontaneously ordered from right-to-left by DO participants, in absence of any training or working memory manipulation. Despite this overlearned ordinal disposition, small quantities result to be still associated to the left and large quantities to the right. Apparently, this result is in contrast with studies showing that spatial associations arise from the ordinal position of items in working memory (e.g., van Dijck & Fias, 2011). However, it is noteworthy that the task used by van Dijck & Fias required to memorize and retrieve the order of a random sequence of numbers, while our tasks required neither to encode nor to retrieve the order of cards in working memory. Hence, order seems to affect spatial associations only when participants are “forced” to encode and retrieve the order itself, otherwise, the effect of magnitude seems to spontaneously emerge (Roth et al. 2025a, b).

Interestingly, in the present study, the order elicited by the context of cards did not reverse the typical small-left/large-right pattern of results, despite the context was embedded in each stimulus. To use the definitions proposed in the taxonomy of situated influences on SNAs (Cipora et al., 2018), the context was manipulated intraexperimentally, at a perceptual level, and unrelatedly to the reading/writing direction. This manipulation is different from others that showed to influence the SNARC effect in previous studies (see for example Bächtold et al., 1998; Mingolo et al., 2021). In those studies, in fact, the context was elicited both by being presented at the beginning of the experiment and through task demands. Despite each stimulus highlights the context, the irrelevance of the context for task demands might indicate that the manipulation used in the present study does not make the context salient enough to reverse the pattern of results. Future studies could clarify the effect of the context’s influence, for instance making participants play cards before the experiment or keeping a picture of the individual order of disposition during the whole experiment.

It remains possible that participants’ processing strategies attenuated the influence of card-specific ordering. Participants may have focused selectively on the corner digits while disregarding other card features, effectively treating the stimuli as isolated numbers rather than integrated card representations. Playing cards present numerical information through both symbolic (corner digits) and non-symbolic (suit arrays) formats, and selective attention to only the symbolic component could have reduced the salience of the card context. This processing strategy would transform the task into standard magnitude classification, causing the observed spatial-numerical associations to reflect the conventional left-to-right number arrangement rather than participants’ card-ordering practices. However, the finding that individual differences in card playing experience systematically modulated SNARC slopes specifically for card stimuli, but not for number stimuli, argues against this interpretation. If participants had simply ignored the card context and processed only the numerical digits, card playing experience should not have differentially affected performance with card versus number stimuli.

An alternative possibility is that, although participants have processed cards in their entirety, the effect of digits could still have prevailed on the context. In this regard, a recent study showed that, when symbolic and non-symbolic numerals are simultaneously displayed, the symbolic values of digits drive SNAs, preventing the spatial association for non-symbolic numerals to emerge (Prpic et al., 2023). This could be controlled by presenting modified cards which only display the suits, by using cards that naturally do not present numerical values (i.e., face cards), by employing a Go/No-go setup in which response is only given to cards with certain suits/colours, or by adopting more game-based task instructions (e.g., “does this card beat 5?“) that may enhance the ecological salience of the card context while maintaining focus on magnitude processing. Finally, it is important to clarify that the described results are limited to the population we tested, namely DO participants. It can be expected that these findings would generalize to other participants who arrange cards in descending order coming from other countries with a left-to-right reading/writing direction.

## Conclusions

Previous literature on the SNARC effect showed that both the order and the magnitude of numbers play a role in this effect, but their specific contribution has not been clarified yet. In this regard, a study by Prpic et al. (2016) showed that order and magnitude are differentially involved in SNARC-like effects depending on task requirements. The present study tried to disambiguate these aspects by using a kind of stimuli that are conceived in opposite orders by different people, namely playing cards. In particular, we tested people who order cards in a descending way, because in these people order and magnitude would elicit opposite representations. Results showed that DO participants exhibited standard SNARC effects for both cards and numbers, with no evidence of reversed spatial-numerical associations at the group level. However, correlation analyses revealed critical individual differences: greater card playing experience was associated with weaker SNARC effects for card stimuli specifically and with larger discrepancies between card and number spatial mappings. These findings indicate that while magnitude-based mapping dominated overall, card-specific ordering exerted measurable influence proportional to individual experience, creating domain-specific attenuation of the standard effect. This pattern suggests that magnitude provides the default mechanism for spatial-numerical associations, while order-based representations emerge when both experiential reinforcement and contextual salience are sufficient.

## Data Availability

Data are available at https://osf.io/eax4j/?view_only=33c03373dac04ec0b7b4d5aa00b23b4d.

## References

[CR1] Bächtold, D., Baumüller, M., & Brugger, P. (1998). Stimulus-response compatibility in representational space. *Neuropsychologia*, *36*(8), 731–735. 10.1016/s0028-3932(98)00002-5 9751438 10.1016/s0028-3932(98)00002-5

[CR2] Bueti, D., & Walsh, V. (2009). The parietal cortex and the representation of time, space, number and other magnitudes. *Philosophical Transactions of the Royal Society B: Biological Sciences*. 10.1098/rstb.2009.0028 10.1098/rstb.2009.0028 PMC2685826 19487186

[CR4] Cipora, K., & Wood, G. (2017). Finding the SNARC Instead of Hunting It: A 20∗20 Monte Carlo Investigation. *Frontiers in Psychology*. 10.3389/fpsyg.2017.01194. 8. 28769840 10.3389/fpsyg.2017.01194 PMC5513957

[CR5] Cipora, K., Patro, K., & Nuerk, H. (2018). Situated Influences on Spatial–Numerical Associations. In T. Hubbard (Ed.), *Spatial Biases in Perception and Cognition* (pp. 41–59). Cambridge University Press. 10.1017/9781316651247.004

[CR3] Cipora, K., van Dijck, J. P., Georges, C., Masson, N., Goebel, S., Willmes, K., Pesenti, M., Schiltz, C., & Nuerk, H. C. (2019). A Minority pulls the sample mean: On the individual prevalence of robust group-level cognitive phenomena – the instance of the SNARC effect. *PsyArXiv*. 10.31234/osf.io/bwyr3

[CR6] Dehaene, S., Bossini, S., & Giraux, P. (1993). The Mental Representation of Parity and Number Magnitude. *Journal of Experimental Psychology*, *122*, 371–396. 10.1037/0096-3445.122.3.371

[CR7] Di Giorgio, E., Lunghi, M., Rugani, R., Regolin, L., Dalla Barba, B., Vallortigara, G., & Simion, F. (2019). A mental number line in human newborns. *Developmental Science*, *22*(6), e12801. 10.1111/desc.12801 30676679 10.1111/desc.12801

[CR8] Eccher, E., Josserand, M., Caparos, S., Boissin, E., Buiatti, M., Piazza, M., & Vallortigara, G. (2025). A left-to-right bias in number-space mapping across ages and cultures. *Nature Communications*, *16*(1), 495. 10.1038/s41467-024-55685-x 39794321 10.1038/s41467-024-55685-x PMC11724025

[CR9] Felisatti, A., Laubrock, J., Shaki, S., & Fischer, M. H. (2020). A biological foundation for spatial–numerical associations: The brain’s asymmetric frequency tuning. *Annals of the New York Academy of Sciences*, *1477*(1), 44–53. 10.1111/nyas.14418 32645221 10.1111/nyas.14418

[CR10] Fias, W. (1996). The Importance of Magnitude Information in Numerical Processing: Evidence from the SNARC Effect. *Mathematical Cognition*, *2*(1), 95–110. 10.1080/135467996387552

[CR11] Fumarola, A., Prpic, V., Pos, D., Murgia, O., Umiltà, M., C., & Agostini, T. (2014). Automatic spatial association for luminance. *Attention Perception & Psychophysics*, *76*(3), 759–765. 10.3758/s13414-013-0614-y 10.3758/s13414-013-0614-y 24402699

[CR12] Gevers, W., Reynvoet, B., & Fias, W. (2003). The mental representation of ordinal sequences is spatially organized. *Cognition*, *87*(3), B87–95. 10.1016/s0010-0277(02)00234-2 12684205 10.1016/s0010-0277(02)00234-2

[CR13] Koch, N. N., Huber, J. F., Lohmann, J., Cipora, K., Butz, M. V., & Nuerk, H. C. (2023). Mental Number Representations Are Spatially Mapped Both by Their Magnitudes and Ordinal Positions. *Collabra: Psychology*, *9*(1), 67908. 10.1525/collabra.67908

[CR14] Lorch, R. F., & Myers, J. L. (1990). Regression analyses of repeated measures data in cognitive research. *Journal of Experimental Psychology: Learning Memory and Cognition*, *16*(1), 149–157. 10.1037/0278-7393.16.1.149 2136750 10.1037//0278-7393.16.1.149

[CR15] Mingolo, S., Prpic, V., Bilotta, E., Fantoni, C., Agostini, T., & Murgia, M. (2021). Snarcing with a phone: The role of order in spatial-numerical associations is revealed by context and task demands. *Journal of Experimental Psychology: Human Perception and Performance*, *47*(10), 1365–1377. 10.1037/xhp0000947 34766820 10.1037/xhp0000947

[CR16] Mingolo, S., Prpic, V., Mariconda, A., Brugger, P., Drack, T., Bilotta, E., Agostini, T., & Murgia, M. (2024). It’s SNARC o’ clock: Manipulating the salience of the context in a conceptual replication of Bächtold et al.’s (1998) clockface study. *Psychological Research Psychologische Forschung*, *88*(3), 837–851. 10.1007/s00426-023-01893-x 37878155 10.1007/s00426-023-01893-x PMC10965594

[CR17] Mingolo, S., Prpic, V., Mariconda, A., Agostini, T., & Murgia, M. (2025). Unravelling the small number bias: The role of pseudoneglect and frequency of use in random number generation. *Psychological Research Psychologische Forschung*, *89*(2), 76. 10.1007/s00426-025-02101-8 40131495 10.1007/s00426-025-02101-8 PMC11937118

[CR18] Murgia, M., Mingolo, S., Prpic, V., Sors, F., Santoro, I., Bilotta, E., & Agostini, T. (2020). University Students’ Hangover May Affect Cognitive Research. *Frontiers in Psychology*. 10.3389/fpsyg.2020.573291. 11. 33132981 10.3389/fpsyg.2020.573291 PMC7550527

[CR19] Peirce, J., Gray, J. R., Simpson, S., MacAskill, M., Höchenberger, R., Sogo, H., Kastman, E., & Lindeløv, J. K. (2019b). PsychoPy2: Experiments in behavior made easy. *Behavior Research Methods*, *51*(1), 195–203. 10.3758/s13428-018-01193-y 30734206 10.3758/s13428-018-01193-y PMC6420413

[CR20] Pitt, B., & Casasanto, D. (2020). The correlations in experience principle: How culture shapes concepts of time and number. *Journal of Experimental Psychology General*, *149*(6), 1048–1070. 10.1037/xge0000696 31633369 10.1037/xge0000696

[CR22] Prpic, V., Fumarola, A., De Tommaso, M., Luccio, R., Murgia, M., & Agostini, T. (2016). Separate mechanisms for magnitude and order processing in the spatial-numerical association of response codes (SNARC) effect: The strange case of musical note values. *Journal of Experimental Psychology Human Perception and Performance*, *42*(8), 1241–1251. 10.1037/xhp0000217 26950384 10.1037/xhp0000217

[CR23] Prpic, V., Mingolo, S., Agostini, T., & Murgia, M. (2021). Magnitude and Order are Both Relevant in SNARC and SNARC-like Effects: A Commentary on Casasanto and Pitt (2019). *Cognitive Science*, *45*(7), e13006. 10.1111/cogs.13006 34213789 10.1111/cogs.13006

[CR21] Prpic, V., Basamh, Y. A., Goodridge, C. M., Agostini, T., & Murgia, M. (2023). Contrasting symbolic and non-symbolic numerical representations in a joint classification task. *Psychonomic Bulletin & Review*. 10.3758/s13423-023-02246-w 10.3758/s13423-023-02246-w PMC10482780 36650364

[CR24] Restle, F. (1970). Speed of adding and comparing numbers. *Journal of Experimental Psychology*, *83*(2), 274–278. 10.1037/h0028573

[CR25] Roth, L., Caffier, J., Reips, U. D., Nuerk, H. C., Overlander, A. T., & Cipora, K. (2025a). One and only SNARC? Spatial-Numerical Associations are not fully flexible and depend on both relative and absolute number magnitude. *Royal Society Open Science*, *12*(1), 241585. 10.1098/rsos.241585 39780972 10.1098/rsos.241585 PMC11709453

[CR26] Roth, L., Huber, J. F., Kronenthaler, S., van Dijck, J. P., Cipora, K., Butz, M. V., & Nuerk, H. C. (2025b). Looks like SNARC spirit: Coexistence of short- and long-term associations between letters and space. *Quarterly Journal of Experimental Psychology*, *78*(10), 2110–2132. 10.1177/17470218251324437 10.1177/17470218251324437 PMC12432287 39960047

[CR27] Rugani, R., Kelly, D. M., Szelest, I., Regolin, L., & Vallortigara, G. (2010). Is it only humans that count from left to right? *Biology Letters*, *6*(3), 290–292. 10.1098/rsbl.2009.0960 20071393 10.1098/rsbl.2009.0960 PMC2880063

[CR28] Rugani, R., Vallortigara, G., Priftis, K., & Regolin, L. (2015). Number-space mapping in the newborn chick resembles humans’ mental number line. *Science*, *347*(6221), 534–536. 10.1126/science.aaa1379 25635096 10.1126/science.aaa1379

[CR29] Rugani, R., Vallortigara, G., Priftis, K., & Regolin, L. (2020). Numerical magnitude, rather than individual bias, explains spatial numerical association in newborn chicks. *eLife*, *9*, e54662. 10.7554/eLife.54662 32584257 10.7554/eLife.54662 PMC7316507

[CR30] Sellaro, R., Treccani, B., Job, R., & Cubelli, R. (2015). Spatial coding of object typical size: Evidence for a SNARC-like effect. *Psychological Research Psychologische Forschung*, *79*(6), 950–962. 10.1007/s00426-014-0636-7 25476998 10.1007/s00426-014-0636-7

[CR31] Shaki, S., & Fischer, M. H. (2008). Reading space into numbers – a cross-linguistic comparison of the SNARC effect. *Cognition*, *108*(2), 590–599. 10.1016/j.cognition.2008.04.001 18514179 10.1016/j.cognition.2008.04.001

[CR32] Shaki, S., & Fischer, M. H. (2014). Random walks on the mental number line. *Experimental Brain Research*, *232*(1), 43–49. 10.1007/s00221-013-3718-7 24091774 10.1007/s00221-013-3718-7

[CR33] Shaki, S., Fischer, M. H., & Petrusic, W. M. (2009). Reading habits for both words and numbers contribute to the SNARC effect. *Psychonomic Bulletin & Review*, *16*(2), 328–331. 10.3758/PBR.16.2.328 19293102 10.3758/PBR.16.2.328

[CR34] Toomarian, E. Y., & Hubbard, E. M. (2018). On the genesis of spatial-numerical associations: Evolutionary and cultural factors co-construct the mental number line. *Neuroscience and Biobehavioral Reviews*, *90*, 184–199. 10.1016/j.neubiorev.2018.04.010 29684402 10.1016/j.neubiorev.2018.04.010 PMC5993626

[CR35] van Dijck, J. P., & Fias, W. (2011). A working memory account for spatial-numerical associations. *Cognition*, *119*(1), 114–119. 10.1016/j.cognition.2010.12.013 21262509 10.1016/j.cognition.2010.12.013

[CR36] Walsh, V. (2003). A theory of magnitude: Common cortical metrics of time, space and quantity. *Trends in Cognitive Sciences*, *7*(11), 483–488. 10.1016/j.tics.2003.09.002 14585444 10.1016/j.tics.2003.09.002

[CR37] Wood, G., Nuerk, H. C., & Willmes, K. (2006). Crossed Hands and the SNARC Effect: A failure to Replicate Dehaene, Bossini and Giraux (1993). *Cortex; A Journal Devoted To The Study Of The Nervous System And Behavior*, *42*(8), 1069–1079. 10.1016/S0010-9452(08)70219-3 17209413 10.1016/s0010-9452(08)70219-3

